# Examining the relationship between cardiac troponin T and cardiac morphology, function, and fibrosis - a cardiac magnetic resonance study

**DOI:** 10.1186/1532-429X-17-S1-Q116

**Published:** 2015-02-03

**Authors:** Mohamad G Ghosn, Stephen Pickett, Patrick W Green, Eric Y Yang, Vijay Nambi, Christie M Ballantyne, Dipan J Shah

**Affiliations:** 1 Baylor College of Medicine, Houston, TX, USA; 2 Cardiology, Baylor College of Medicine, Houston, TX, USA; 3 Cardiology, Houston Methodist DeBakey Heart & Vascular Center, Houston, TX, USA

## Background

Elevated high sensitivity cardiac troponin (cTnT) levels, LVEF, and the degree of myocardial replacement fibrosis (RF) have been shown to be significantly associated with cardiovascular outcomes. However, the relationship amongst them has yet to be studied. Cardiac magnetic resonance (CMR) has become the gold standard for viability imaging and accurately characterizing RF. Recently, a new technique has been employed to quantify diffuse myocardial fibrosis via assessment of myocardial extracellular volume (ECV). The current study sought to investigate the association between ECV, RF, LVEF, LV mass (LVM) and cTnT levels.

## Methods

We enrolled 192 patients who underwent cine CMR for assessment of LVM and LVEF, delay enhancement CMR (DE-CMR) for assessment of RF, and T1 mapping for assessment of diffuse interstitial fibrosis (ECV). Patients with hypertrophic cardiomyopathy were excluded. Serum samples collected at time of scan were assayed for cTnT levels. The extent of RF was determined by visual inspection. ECV was estimated from the concentration of extracellular contrast agent in the myocardium relative to the blood in the dynamic steady state. Care was taken to avoid measuring ECV in regions with RF detected on DE-CMR.

## Results

Mean age of enrolled patients was 62±14 years (59% males) with an average LVEF of 58±16%. RF in the LV myocardium was present in 84 patients (41%). The mean cTnT value was 27ng/L and the median (interquartile range; IQR) was 11 ng/L (6 ng/L, 20 ng/L). Using Kruskal-Wallis test with TnT quartiles as cuttoffs, ECV showed a non-significant positive relationship with increased levels of Troponin (Χ^2^= 3.0). With the same statistical test, the following parameters showed a very significant relationship: RF (Χ^2^=13.8, p<0.01), LVEF (Χ^2^= 21.3, p<0.01), LV end diastolic volume (Χ^2^=10.5, p=0.01), LV mass (Χ^2^=23.1, p<0.01).

## Conclusions

This study demonstrates a significant mean rank between RF, LVEF, and LVM with cTnT levels. It also shows a positive relationship between ECV and cTnT. However, a larger study is needed to elucidate the true relationship between ECV and cTnT.

## Funding

N/A.

**Figure 1:**
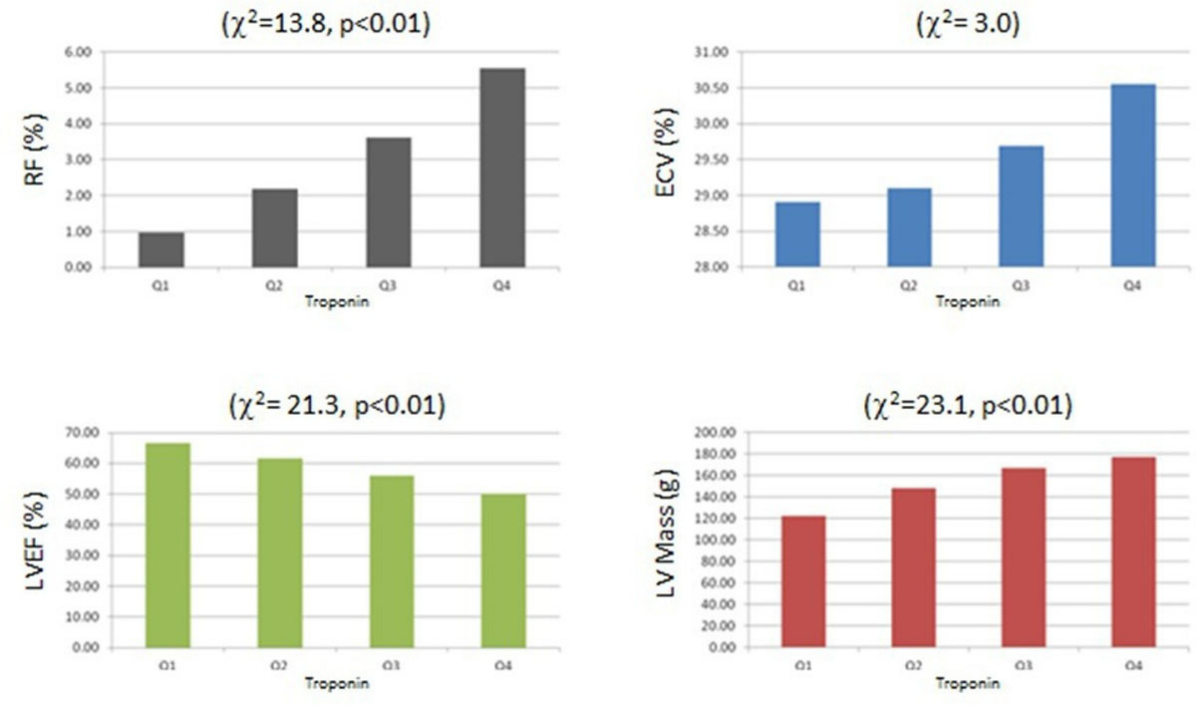
The LV mass and the percentages of RF, ECV, and LVEF according to the Quartile cardiac Troponin (cTnT) Levels.

